# A Clinical-Radiomic Nomogram Based on Unenhanced Computed Tomography for Predicting the Risk of Aldosterone-Producing Adenoma

**DOI:** 10.3389/fonc.2021.634879

**Published:** 2021-07-09

**Authors:** Keng He, Zhao-Tao Zhang, Zhen-Hua Wang, Yu Wang, Yi-Xi Wang, Hong-Zhou Zhang, Yi-Fei Dong, Xin-Lan Xiao

**Affiliations:** ^1^ Department of Radiology, The Second Affiliated Hospital of Nanchang University, Nanchang, China; ^2^ Department of Cardiovascular Medicine, The Second Affiliated Hospital of Nanchang University, Nanchang, China

**Keywords:** adenoma, radiomics, primary aldosteronism, nomogram, precision medicine

## Abstract

**Purpose:**

To develop and validate a clinical-radiomic nomogram for the preoperative prediction of the aldosterone-producing adenoma (APA) risk in patients with unilateral adrenal adenoma.

**Patients and Methods:**

Ninety consecutive primary aldosteronism (PA) patients with unilateral adrenal adenoma who underwent adrenal venous sampling (AVS) were randomly separated into training (n = 62) and validation cohorts (n = 28) (7:3 ratio) by a computer algorithm. Data were collected from October 2017 to June 2020. The prediction model was developed in the training cohort. Radiomic features were extracted from unenhanced computed tomography (CT) images of unilateral adrenal adenoma. The least absolute shrinkage and selection operator (LASSO) regression model was used to reduce data dimensions, select features, and establish a radiomic signature. Multivariable logistic regression analysis was used for the predictive model development, the radiomic signature and clinical risk factors integration, and the model was displayed as a clinical-radiomic nomogram. The nomogram performance was evaluated by its calibration, discrimination, and clinical practicability. Internal validation was performed.

**Results:**

Six potential predictors were selected from 358 texture features by using the LASSO regression model. These features were included in the Radscore. The predictors included in the individualized prediction nomogram were the Radscore, age, sex, serum potassium level, and aldosterone-to-renin ratio (ARR). The model showed good discrimination, with an area under the receiver operating characteristic curve (AUC) of 0.900 [95% confidence interval (CI), 0.807 to 0.993], and good calibration. The nomogram still showed good discrimination [AUC, 0.912 (95% CI, 0.761 to 1.000)] and good calibration in the validation cohort. Decision curve analysis presented that the nomogram was useful in clinical practice.

**Conclusions:**

A clinical-radiomic nomogram was constructed by integrating a radiomic signature and clinical factors. The nomogram facilitated accurate prediction of the probability of APA in patients with unilateral adrenal nodules and could be helpful for clinical decision making.

## Introduction

Primary aldosteronism (PA) is a common cause of secondary hypertension, and the PA prevalence according to hypertension stage is as follows: stage 1, 1.99%; stage 2, 8.02%; and stage 3, 13.2% (1). In patients with resistant hypertension, the prevalence rate is higher, nearly 17–23% (2–5). PA patients have more severe cardiovascular and renal outcomes than essential hypertension (EH) patients with the same blood pressure (6–9). Aldosterone-producing adenoma (APA) is one of the most common types of PA and is mainly treated by surgical resection of lesions with excessive aldosterone secretion (10).

In 2008, the Endocrine Society began to recommend the use of adrenal venous sampling (AVS) as a method of localized diagnosis of PA (11). AVS is the gold standard test for defining the PA subtype. However, AVS is technically difficult to conduct because of its complicated method and high cost; currently, only a few medical centers have adopted this mature technology (12). Previous studies have suggested that adrenal computed tomography (CT) cannot accurately diagnose PA (13). With the development of high-throughput computing, countless quantitative features that can be rapidly extracted from tomographic images, such as CT images. The process of transforming digital medical images into high-dimensional data that can be mined is known as radiomics (14, 15). Quantitative analyses can reveal a correlation between biomedical images and potential pathophysiology. Radiomic data contain first-order, second-order, and higher-order statistics. These data, combined with other relevant patient data, can be used as complex bioinformatics mining tools to develop models that may improve the accuracy of diagnosis, prognosis, and prediction (16, 17). To the best of our knowledge, there are no studies that have determined whether radiomic signatures can predict the risk of APA.

Therefore, the purpose of this study was to develop and validate a radiomic nomogram combining with both the radiomics signature and clinical risk factors to predict the risk of APA in different individuals.

## Materials and Methods

### Patients

**Figure 1:**
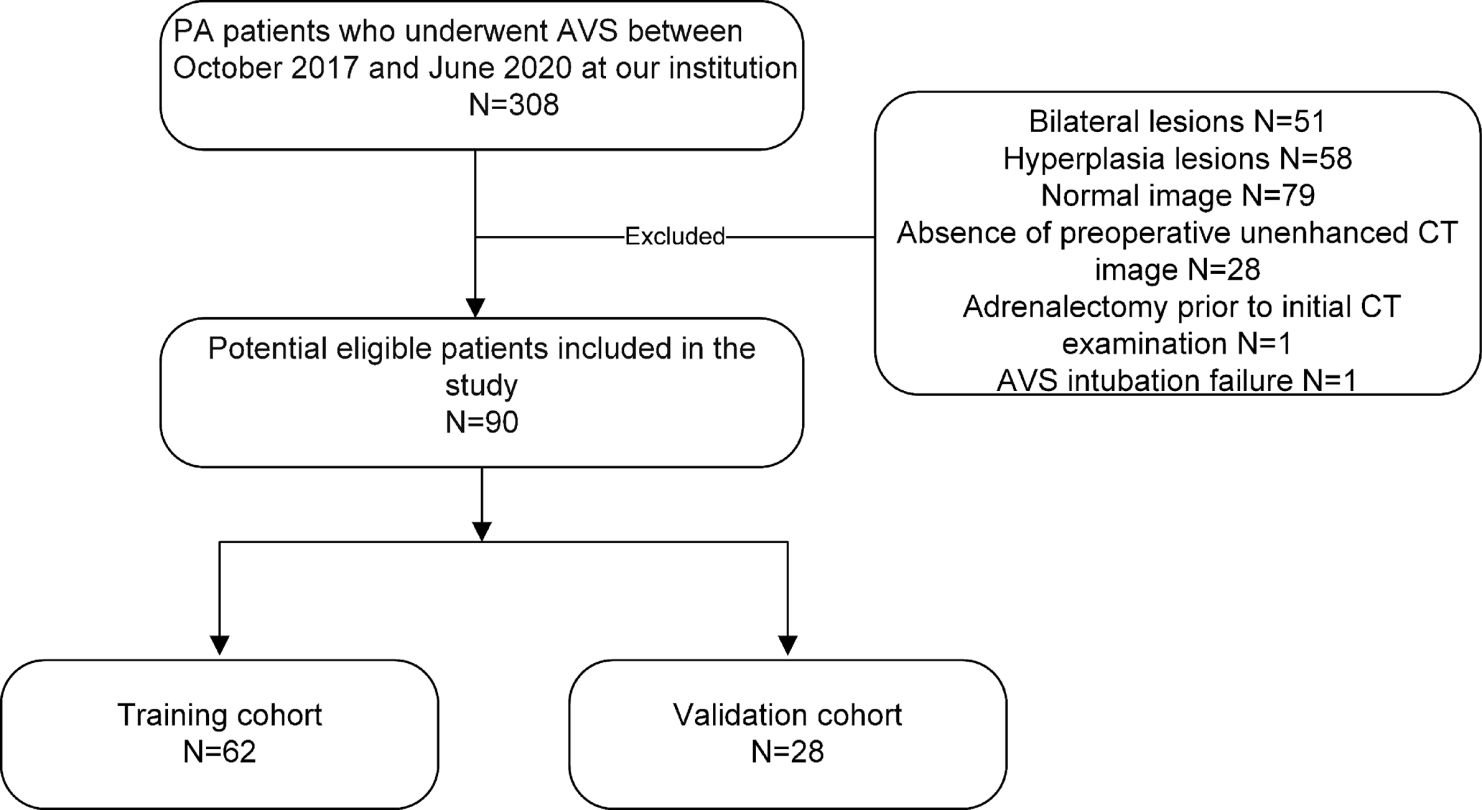
The patient enrollment pathway, along with the inclusion and exclusion criteria.

Our study recruited 90 consecutive PA patients (38 female and 52 male patients; mean age, 50.58 ± 10.62 years; age range, 26–74 years) with unilateral adrenal adenoma who underwent AVS between October 2017 and June 2020 by searching our electronic hospital information system. The patients were included based on the following inclusion criteria: 1) biochemical diagnosis of PA in agreement with the Endocrine Society guideline (10); 2) presence of a unilateral adrenal adenoma on CT before AVS; 3) availability of clinical characteristics; and 4) completion of CT image datasets and medical history at our hospital. The exclusion criteria were as follows: 1) adrenalectomy prior to initial CT examination; 2) AVS intubation failure [defined as a selectivity index (SI)] (the ratio of adrenal and peripheral cortisol) <2.0) (10); and 3) severe respiratory artifacts on CT images. The patients were randomly separated into training (n = 62) and validation cohorts (n = 28) (7:3 ratio) by a computer algorithm. APA was diagnosed in the patients based on the following criteria: 1) a lateralization index (LI) >2.0 at AVS; 2) a unilateral adrenal nodule on CT consistent with the LI; and 3) a blood pressure decreases after adrenalectomy or adrenal artery embolization. **Figure 1** consists of a flow chart that shows the patient recruitment pathway.

### Image Acquisition and Analysis

CT examinations were performed using the following multidetector CT scanners: SOMATOM Definition Flash (Siemens Healthcare) and Brilliance (Philips Healthcare). The scanning parameters were as follows: 100–120 kV, 200–250 mA, and 2**-**5 mm slice thickness. All CT images were preprocessed before region of interest (ROI) segmentation and radiomic feature extraction by using AK software (Artificial Intelligence Kit V3.0.0.R, GE Healthcare). Resampling and gray intensity normalization were performed to eliminate the heterogeneity of the CT scan parameters (18). Images were resampled to 1 × 1 × 1 mm³ voxels using linear interpolation. The gray levels of all CT images were then normalized into 0 to 255.

Adrenal adenomas were diagnosed on thin-sliced unenhanced CT images by two radiologists (one had 6 years of experience in abdominal radiology and the other had 10 years of experience.). A typical unilateral adenoma was defined as a unilateral hypodense nodule [in unenhanced CT mean attenuation value <18 Hounsfield units (HU) and minimum attenuation value <0 HU; the details are shown in the in the **Supplementary Table 1**]. At least 10 mm in diameter, while the other ipsilateral and contralateral glands were smooth and free from hyperplasia. All adrenal nodules were verified by a consensus of two radiologists. If the diagnosis opinions were inconsistent, a professor of radiology who had 37 years of experience in abdominal radiology was consulted.

### Intraobserver and Interobserver Agreement

The intraobserver and interobserver agreements for feature extraction were assessed by the intraclass correlation coefficient (ICC). At first, 20 random CT images were selected for ROI segmentation and feature extraction. Two experienced radiologists completed the ROI segmentation independently. The intraobserver ICC was calculated by comparing the two feature extractions made by reader A (with 6 years of experience in abdominal radiology). The interobserver ICC was calculated by comparing the first feature extraction performed by reader A with the feature extractions conducted by another reader (reader B, with 10 years of experience in abdominal radiology). Features with values greater than 0.75 for both the interobserver ICC and the intraobserver ICC were included in the analysis, and the rest of image segmentations were completed by reader A.

### ROI Segmentation and Radiomic Feature Extraction

The ROI covering the entire surface of the lesion on each consecutive slice was manually segmented with ITK-SNAP software (version 3.8.0; www.itksnap.org). Then, the ROI was matched with the corresponding CT images using AK software (Artificial Intelligence Kit V3.0.0.R, GE Healthcare) to extract the radiomic features.

### Feature Selection and Radiomic Signature Construction

The LASSO method (19), which is suitable for dimensionality reduction of high-dimensional data (20), was used for the selection of the optimal predictive features from the radiomic features. A radiomic score (Radscore) was computed for each patient *via* a multiple logistic regression formula.

$$Radscore=1÷\left(1+e^{-\left(\beta_{0}+\beta_{1}*X_{1}+\beta_{2}*X_{2}+\cdots +\beta_{m}*X_{m}\right)}\right)$$

### Development of the Clinical-Radiomic Model and Nomogram

The LASSO method was used for the selection of the optimal predictive features from the training cohort. To avoid overfitting, five cross-validations were performed. Five selected variables [age, sex, serum potassium, Radscore, and aldosterone-to-renin ratio (ARR)] were incorporated into the multiple logistic regression to construct the clinical-radiomic model. Validation was performed in the validation cohort. At the end, this model was developed into a clinical-radiomic nomogram, and calibration plots were used to examine the performance characteristics of the nomogram in detail.

### Statistical Analysis

The statistical analysis was performed using R software (version 3.6.2; http://www.Rproject.org). The data distribution of the data was evaluated by the Kolmogorov–Smirnov test. Normally distributed variables were compared using Student’s t-test and are described as the mean [standard deviation (SD)]. Non-normally distributed variables were analyzed by the Mann–Whitney’s test and are described as medians [interquartile ranges (IQRs)]. Categorical variables were analyzed by the chi-square test or Fisher’s exact test and are described as absolute numbers (n) and proportions (%). All statistical tests were two-sided, and P-values of <0.05 were regard as significant.

## Results

### Patients’ Characteristics

There were no significant differences in the variables [Radscore, age, sex, serum potassium, estimated glomerular filtration rate (eGFR), systolic blood pressure (SBP), diastolic blood pressure (DBP), body mass index (BMI), ARR and APA] between the training and validation cohorts (**Table 1**, P > 0.05), indicating that the use of random seeds to randomly group the total data was reasonable. There were significant differences in variables, including the Radscore and serum potassium level between APA-positive and APA-negative patients in the training and validation cohorts (**Table 2**, P < 0.05).

**Table 1:** Clinical characteristics of the training and validation cohorts.

Variable	Whole cohort	Training cohort	Validation cohort	P-value
	*N = 90*	*N = 62*	*N = 28*	
Radscore	0.69 [0.64;0.74]	0.69 [0.64;0.73]	0.68 [0.64;0.76]	0.708
age (years)	50.58 (10.62)	49.82 (9.40)	52.25 (12.97)	0.379
sex:				0.882
Male	52 (57.78%)	35 (56.45%)	17 (60.71%)	
Female	38 (42.22%)	27 (43.55%)	11 (39.29%)	
serum potassium (mmol/L)	3.38 (0.60)	3.36 (0.63)	3.41 (0.54)	0.702
eGFR (ml/min/1.73 m^2^)	92.32 (24.01)	93.40 (23.73)	89.92 (24.88)	0.537
SBP (mmHg)	152.79 (22.64)	151.55 (23.04)	155.54 (21.86)	0.434
DBP (mmHg)	92.56 (16.81)	93.23 (17.10)	91.07 (16.35)	0.571
BMI (kg/m^2^)	24.97 (3.50)	25.08 (3.74)	24.73 (2.95)	0.631
ARR (ng/dl)/(ng/ml/h)	1068.00 [249.77;3019.88]	1354.00 [220.86;2999.25]	972.50 [259.53;3015.38]	0.868
APA:				0.458
no	29 (32.22%)	22 (35.48%)	7 (25.00%)	
yes	61 (67.78%)	40 (64.52%)	21 (75.00%)	

Normally and non-normally distributed variables are presented as mean (SD) or median [IQR], as appropriate. Categorical variables are presented as absolute number (n) and proportion (%).

APA, Aldosterone-Producing Adenoma; eGFR, estimated Glomerular Filtration Rate; SBP, Systolic Blood Pressure; DBP, Diastolic Blood Pressure; BMI, Body Mass Index; ARR, Aldosterone-to-Renin Ratio.

**Table 2:** Clinical characteristics of APA-positive and APA-negative patients in the training and validation cohorts.

Variable	Training cohort		P-Value	Validation cohort		P-Value
	Non-APA	APA		Non-APA	APA	
	*N = 22*	*N = 40*		*N = 7*	*N = 21*	
Radscore	0.63 [0.60;0.69]	0.70 [0.66;0.74]	0.001	0.59 [0.53;0.68]	0.71 [0.66;0.76]	0.036
age(years)	52.23 (6.54)	48.50 (10.49)	0.091	57.00 (8.37)	50.67 (13.98)	0.167
sex:			0.304			1.000*
Male	10 (45.45%)	25 (62.50%)		4 (57.14%)	13 (61.90%)	
Female	12 (54.55%)	15 (37.50%)		3 (42.86%)	8 (38.10%)	
serum potassium (mmol/L)	3.60 (0.52)	3.23 (0.65)	0.017	3.76 (0.26)	3.30 (0.56)	0.007
eGFR (ml/min/1.73 m^2^)	92.27 (18.73)	94.02 (26.29)	0.762	101.20 (30.06)	86.17 (22.47)	0.258
SBP (mmHg)	155.77 (26.92)	149.22 (20.61)	0.328	152.29 (23.99)	156.62 (21.63)	0.681
DBP (mmHg)	97.32 (20.54)	90.97 (14.67)	0.210	85.57 (16.31)	92.90 (16.34)	0.327
BMI (kg/m^2^)	25.48 (2.96)	24.87 (4.13)	0.503	23.49 (3.84)	25.14 (2.57)	0.321
ARR (ng/dl)/(ng/ml/h)	928.50 [132.87;2225.75]	1538.83 [506.80;3193.25]	0.085	559.33 [501.75;834.50]	1084.00 [246.89;3375.00]	0.442

Normally and non-normally distributed variables are presented as mean (SD) or median [IQR], as appropriate. Categorical variables presented as absolute number (n) and proportion (%).

APA, Aldosterone-Producing Adenoma; eGFR, estimated Glomerular Filtration Rate; SBP, Systolic Blood Pressure; DBP, Diastolic Blood Pressure; BMI, Body Mass Index; ARR, Aldosterone-to-Renin Ratio.

*Fisher’s exact test.

### Interobserver and Intraobserver Reproducibility of Radiomic Feature Extraction

The intraobserver ICC computed based on two measurements of reader A ranged from 0.753 to 0.999 (median 0.962, IQR 0.910–0.989). The interobserver agreement between the two readers ranged from 0.751 to 0.998 (median 0.941, IQR 0.893–0.980). The results showed favorable intraobserver and interobserver feature extraction reproducibility.

### Radiomic Signature Construction

Six potential predictors were selected from 358 texture features by using the LASSO regression model. These features were included in the Radscore, which is calculated by using the following formula: 

Radscore=1÷(1+e^(−(14.83134699 +ShortRunLowGreyLevelEmphasis_AllDirection_offset4_SD ∗(−5276158337)+ShortRunHighGreyLevelEmphasis_angle135_offset1∗(−0.00023976)+ClusterProminence_angle90_offset7∗(−0.0000457)+ kurtosis*0.058185896+LongRunEmphasis_AllDirection_offset7_SD∗0.228221296+ GLCMEnergy_angle90_offset7∗1.887620474) )).

The Radscore was computed for each patient using this formula. (The details of model are shown in the attached file in **Supplementary Data 1** and **Supplementary Figure 1**).

### Development of the Clinical-Radiomic Model and the Clinical-Radiomic Nomogram

**Figure 2:**
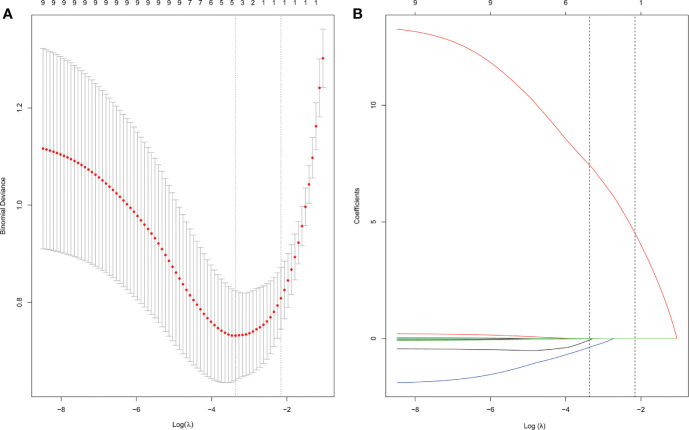
APA candidate variable selection using LASSO regression. **(A)** Binomial deviation graph of the optimal tuning parameter (*λ*) in the LASSO model. **(B)** LASSO coefficient profiles of the nine possible influencing factors.

**Figure 3:**
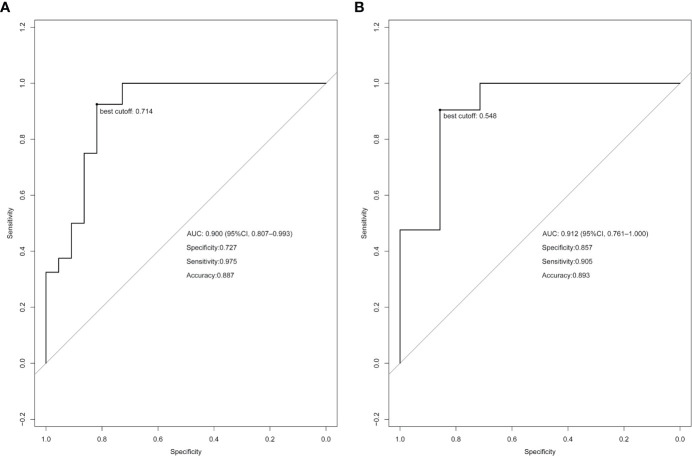
Receiver operating characteristic (ROC) curve analysis based on the model prediction. The best cutoff values are indicated on the curves. **(A)** ROC curve of the training cohort. **(B)** ROC curve of the validation cohort.

**Figure 4:**
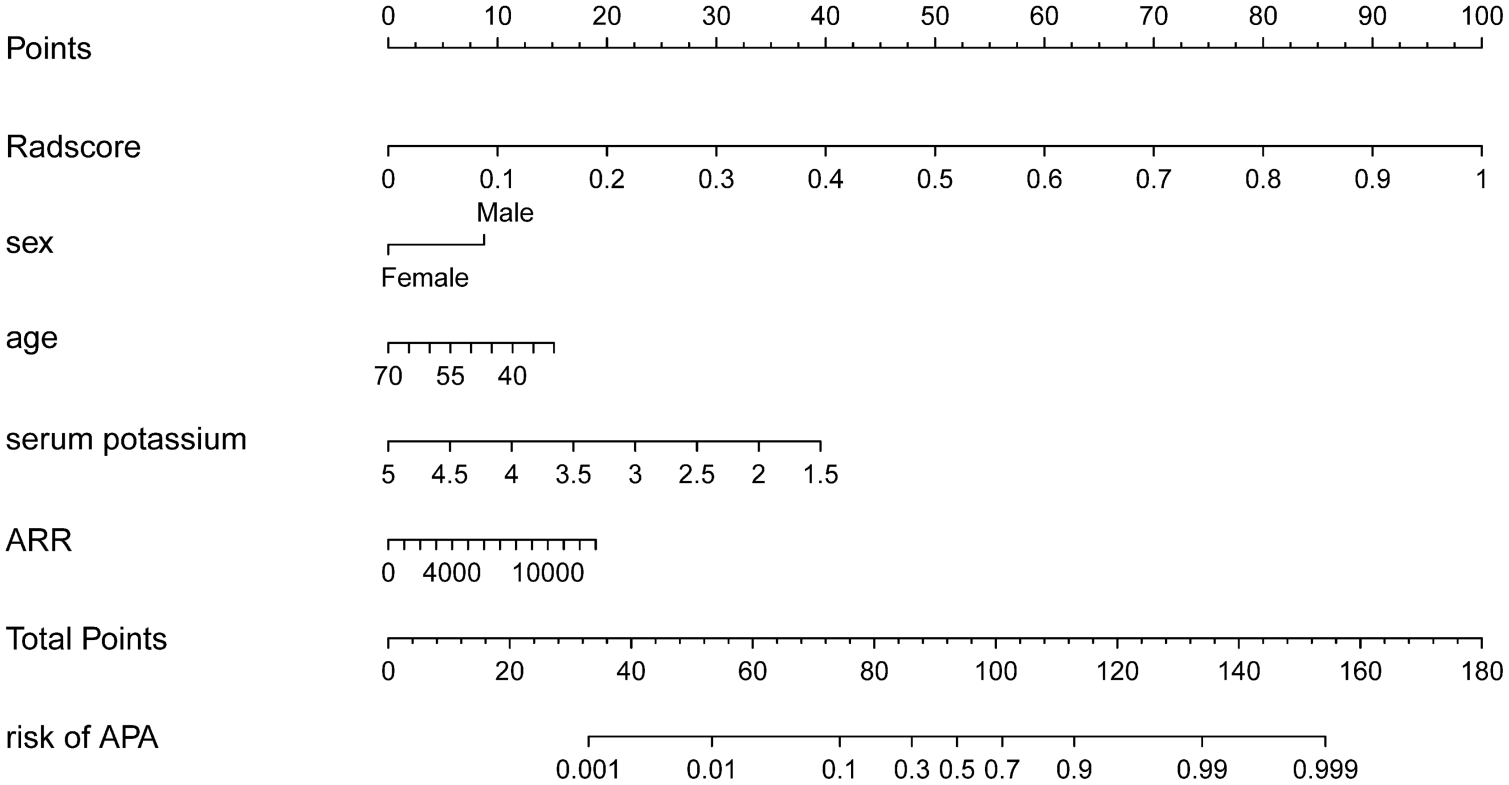
The clinical-radiomic nomogram that was developed.

**Figure 5:**
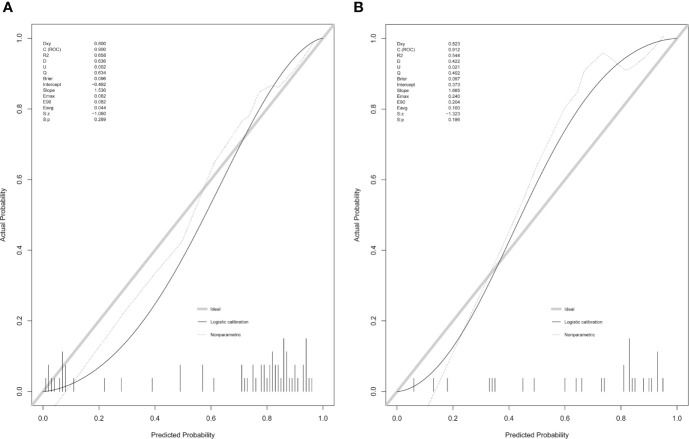
Calibration curves of the clinical-radiomic nomogram in the training cohort **(A)** and validation cohort **(B)**.

Using the LASSO regression model, five selected variables (age, sex, serum potassium, Radscore, and ARR) were incorporated into the multiple logistic regression to construct the clinical-radiomic model (**Figures 2A, B**). The clinical-radiomic model showed good predictive efficacy, with an AUC of 0.900 [95% confidence interval (CI), 0.807 to 0.993] in the training cohort and 0.912 (95% CI, 0.761 to 1.000) in the validation cohort (**Figures 3A, B**). A clinical-radiomic nomogram including these five predictors was built (**Figure 4**). Non-significant values of the unreliability U test statistic (P = 0.289) indicated good calibration in the training cohort and the validation cohort (P = 0.186) (**Figures 5A, B**).

### Clinical Utility According to Decision Curve Analysis

**Figure 6:**
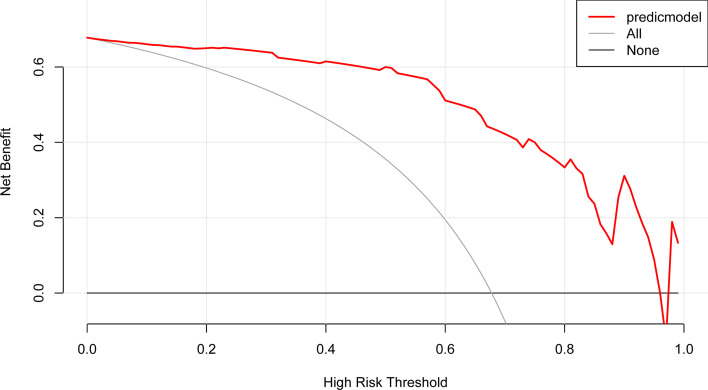
DCA of the nomogram model. The y-axis represents the net benefit. The red line represents the predictive APA nomogram model. The gray line represents the assumption that all patients have APA. The black line represents the assumption that no patients have APA.

The DCA of the clinical-radiomic model is displayed in **Figure 6**. The clinical-radiomic nomogram exhibited the most clinical usefulness across nearly all of the threshold probabilities, indicating that the nomogram is a reliable clinical treatment tool for predicting the risk of APA in PA patients with unilateral adrenal adenoma.

## Discussion

To the best of our knowledge, few studies have developed a clinical-radiomic nomogram based on radiomic analysis that combines crucial clinical risk factors to predict the risk of APA in patients with unilateral adrenal adenoma. Various clinical risk factors have been included in predictive models for the subtype diagnosis of PA. Küpers et al. found that serum potassium levels <3.5 mmol/L and an eGFR <100 ml/min/1.73 m^2^ were clinical risk factors for PA (21). Other researchers have found that serum potassium, plasma aldosterone, ARR, and sex were clinical risk factors for bilateral hyperaldosteronism (22–24). Another study found that age of onset of hypertension and serum potassium were independently related to the PA (25). Failure to clearly define the dominant side of aldosterone secretion is the major limitation of all others previously proposed factors. Similarly, age, sex, serum potassium, and ARR were the clinical features used to predict APA in our study, but our model can be used to predict the dominant side of aldosterone secretion for PA patients with unilateral adrenal adenoma.

Compared with the published studies focusing on the risk factors related to APA, this study not only evaluates the clinical features but also attempts to mine additional information from the radiomic features extracted from the CT images by machine learning. The radiomic features include not only the first-order features (such as histogram parameters and texture parameters) but also the higher-order features [such as the gray level co-occurrence matrix (GLCM) and gray level run-length matrix (RLM)] that cannot be captured by the human visual system but can be reflected by radiomic analysis. Radiomics has attracted more and more attention because of its potential to build predictive models that associate image features with phenotypes or gene signatures (16, 17). A study by the Mayo Center found that using imaging-based classification strategies alone resulted in 20% of patients not undergoing adrenalectomy, 15 to 25% of patients undergoing unnecessary adrenalectomy, and 4% of patients undergoing the incorrect removal of the adrenal gland (13). Another study of CT scans showed that 28.3% of adrenal macroadenomas were hyperplastic, of which nephrectomy failed to improve blood pressure in 66.7% (26). To the best of our knowledge, no previously published study has determined whether radiomics can be used to distinguish APA. A study used radiomics to differentiate between lipid-poor adrenal adenoma in adrenal incidentaloma and subclinical pheochromocytoma with high accuracy, sensitivity, and specificity (27). In our study, the prediction results by using radiomic and clinical factors indicated that our method was effective in both the training cohort and the validation cohort, and this model was able to differentiate APA patients from non-APA patients.

The radiomic features obtained in this model include: the histogram parameter feature (kurtosis), which represents the voxel intensity distribution of the adenoma, a texture parameter feature (ClusterProminence_angle90_offset7) that reflects the surface appearance of the adenoma and its element distribution patterns, the GLCM feature (GLCMEnergy_angle90_offset7) and the RLM features (ShortRunLowGreyLevelEmphasis_AllDirection_offset4_SD, ShortRunHighGreyLevelEmphasis_angle135_offset1, LongRunEmphasis_AllDirection_offset7_SD) that reflect the spatial relationships of the adenoma. One study showed that the most significant difference in the GLCM characteristics is between adipose tissue and nucleated tissue, and the GLCM feature is sensitive to the tumor microenvironment (hypoxia) (28). Moreover, another study indicated that features such as GLCM are highly related to the heterogeneity of the tumor vascular structure and function, which may lead to a tumor microenvironment conducive to hypoxia and acidosis (29). Some researchers have found that the RLM features are related to lipid metabolism, axon guidance and synaptic transmission; histogram parameter features are related to biological oxidation and gene expression regulation; and texture features are related to the immune system, neural system, and circadian clock (30). These studies have shown that the radiomic features are related to the biological characteristics of the tumor, and we speculate that these radiomic features in our model may be related to the biological characteristic of secretory function of the adenoma, but the specific mechanism needs to be further investigated.

The adrenal nodule images used in this study were all obtained from unenhanced CT images. Considering that adenomas are easily confused with nodular hyperplasia or hyperplastic nodules, we measured the attenuation value of each nodule individually. The results showed that the minimum attenuation value of each selected nodule was negative (ranging from −59.3 to −10.3 HU), suggesting that the nodule contained fatty components, and the mean attenuation value ranged from −17.2 to 12.0 HU. It has been reported that the mean and minimum attenuation values can be used to identify adenomas, and our results are consistent with these reports (31, 32). Our analysis is limited to unenhanced CT images because we aimed to determine whether this analysis based on unenhanced CT images alone was adequate to make an acceptable distinction. If this analysis is indeed effective, it will help avoid unnecessary enhanced CT scans. In one study, significant texture differences were found in unenhanced scan CT images of lung cancer (33). Studies have shown that voxel size resampling and gray level normalization of images obtained by different CT scanners can obtain reproducible CT features (18, 34). In our study, we performed voxel size resampling and gray level normalization of CT images before extracting image features from different CT scanners in our institution to reduce bias and variance.

Our study had several limitations. First and foremost, this nomogram is based on a single-institution retrospective analysis and may not be applicable to other populations of patients with unilateral adrenal adenoma. Nevertheless, through the analysis of this cohort, a primary clinical-radiomic nomogram was established and can be improved in the future in a bigger and more various prospective study. Furthermore, the model constructed in this study lacks of the external validation. Multicenter validation with a bigger sample size must be performed. Regardless of these limitations, we hope that our experience will contribute to accurate detection of APA. We intend to carry out the next prospective study to further verify and improve our model.

In conclusion, a radiomic nomogram was built by combining clinical factors with a radiomic signature; this nomogram helped to accurately detect the probability of APA in patients with unilateral adrenal adenoma and may aid physicians in clinical decision making.

## Data Availability Statement

The raw data supporting the conclusions of this article will be made available by the authors without undue reservation.

## Ethics Statement

The studies involving human participants were reviewed and approved by the Second Affiliated Hospital of Nanchang University Medical Research Ethics Committee. The ethics committee waived the requirement of written informed consent for participation.

## Author Contributions

Writing original draft preparation and design: KH. Acquisition of data: ZTZ, ZHW, YW, YXW, and HZZ. Analysis and interpretation of data: ZTZ and ZHW. Funding acquisition: ZTZ, HZZ, YFD, and XLX. Revision of the manuscript: YFD and XLX. All authors contributed to the article and approved the submitted version.

## Funding

This study was supported by the National Natural Science Foundation of China (grant number 81960088 and 82060557), the Key Research & Development Program of Jiangxi Province, China (NO. 20171ACG70002) and Graduate Research and Innovation Projects of Jiangxi Province, China (NO. YC2020-B055).

## Conflict of Interest

The authors declare that the research was conducted in the absence of any commercial or financial relationships that could be construed as a potential conflict of interest.
