# Supplementary material for: A Clinical-Radiomic Nomogram Based on Unenhanced Computed Tomography for Predicting the Risk of Aldosterone-Producing Adenoma
Source: Front Oncol. 2021 Jul 9;11:634879. doi: 10.3389/fonc.2021.634879 (PMC8300014; doi:10.3389/fonc.2021.634879)
Supplement: Supplementary file 1 [file DataSheet_1.docx]

Development of the radiomic model and the radiomic nomogram

By using the LASSO regression model, six selected variables (ShortRunLowGreyLevelEmphasis_AllDirection_offset4_SD, ShortRunHighGreyLevelEmphasis_angle135_offset1, ClusterProminence_angle90_offset7,kurtosis,LongRunEmphasis_AllDirection_offset7_SD,GLCMEnergy_angle90_offset7) were incorporated into the multiple logistic regression to construct the radiomic model (Supplementary Fig. 1 A and B). The radiomic model showed advantageous predictive efficacy, with an AUC of 0.885 (95% CI, 0.785 to 0.985) in the training cohort and 0.878 (95% CI, 0.701 to 1.000) in the validation cohort (Supplementary Fig. 1 E and F). A non-significant unreliability U test statistic (P = 0.056) presented good calibration in the training cohort and the validation cohort (P = 0.091) (Supplementary Fig. 1 C and D). A radiomic nomogram including these six predictors was built (Supplementary Fig. 1 G).
