# Supplementary figures and images for: A Clinical-Radiomic Nomogram Based on Unenhanced Computed Tomography for Predicting the Risk of Aldosterone-Producing Adenoma
Source: Front Oncol. 2021 Jul 9;11:634879. doi: 10.3389/fonc.2021.634879 (PMC8300014; doi:10.3389/fonc.2021.634879)

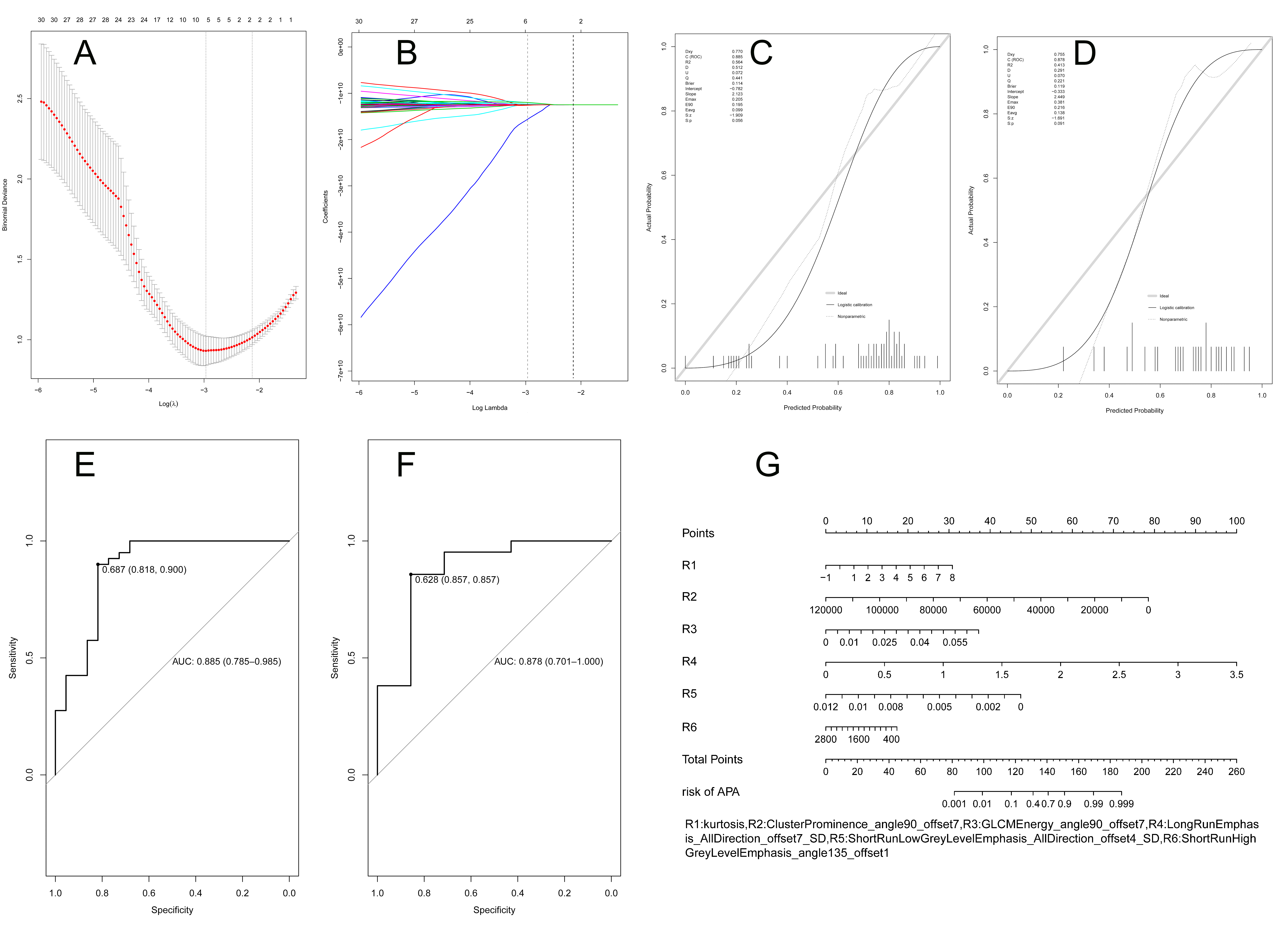

Supplement: Supplementary file 2 [file Image_1.tif]
